# Queer Remedies

**Published:** 1873-01

**Authors:** 


### QUEER REMEDIES.

Foreigners, after arriving in this country, mastering the bewilderments of our language, then reading the patent medicine advertisements to be found displayed in every newspaper and magazine, as well as emblazoned in grotesque and luminous letters upon every rock, tree and fence, possessing area sufficient for the peculiar and gigantic style of chirography adopted by dealers in these mysterious compounds, we should think would at once abandon the idea of settling among, what they would naturally suppose to be, a race of invalids or lunatics—perhaps both. The country is flooded with patent medicine literature; pamphlets, books, lithographs, circulars, signs, handbills, cards and symbols, stare you in the face, and are thrust under your nose at every conceivable time and place.

Dyspepsia prevails in this country to a greater extent than any other; in fact, we are known abroad as a nation of dyspeptics. The curious and innumerable complications and symptoms that are directly attributable to indigestion, are precisely those that most baffle our practitioners of medicine. The treatment of dyspepsia is recognized to be, by all physicians worthy of the name, rather hygeinic than medicinal; and, as nine-tenths of all sick people prefer medicine to advice, and will cheerfully pay for the former and abuse you for the latter, why, the majority of doctors, having an eye single to business, prescribe medicines, allow the patient to pursue his own course with regard to eating, the result of all of which is, a disconsolate and discouraged patient, and a discharged doctor.

It is just at this point that the clarivoyant and other equally ridiculous quacks, are called to the rescue, and when they have surfeited the really-to-be-pitied sufferer, with gin or physic, according to his faith or credulence, then the patent medicine list is gone through with, swelling the enormous gains of these versatile dealers in bitter barks and poor whiskey.

Ever since the beginning of time, it seems, that mortal man has been given to the taking of marvelous compounds for the cure of real or imaginary disease. It is recorded that James I., who doted upon his wisdom, was in the habit of taking a certain elixir from an old quack, who said it would make him "ailment proof." An ancient Duke of Burgundy was fool enough to pay a vast fortune for a balsam to make his memory transcendently good. Several kings and monarchs of ancient times were in the habit of taking all sorts of vile compounds; powdered wood-lice were taken for asthma, and, in some instances were enveloped in paper and swallowed alive. Pliny recommended wood-lice and green lizards, boiled down to a thick soup, then swallowed, for paralysis. Lice, "large lice, begot of your own dust," were taken for jaundice. Walton thought so highly of this remedy that he exclaimed, that heaven itself must have revealed it to the Jews. Bugs were once recommended for hysteria. They were dried, powdered, and taken in water.—Common ants, distilled in spirits of wine, were reputed of great avail in "stirring up a man's magnanimity." Lupton says: "Three nails made on the vigil of St. John, and driven so deep that they cannot be seen, in the place where the party doth fall that hath the falling sickness, and naming the party's name while it is doing, doth drive away the disease quite."—Toothache was formerly cured by digging up a plant with a tool having no iron on it, touching the tooth four times with it, and being careful to spit three times after each touch. Plague and poison were set at naught by wearing "two figs on thy bellie, and two walnuts on thy backe." Warts were banished (and are *now* by many noodles,) by rubbing a piece of *stolen* bacon upon them, and then burying it under the eaves of the house.

Here is a recipe for an ointment, to which was attributed more virtue than that of "Holloway's," of our day: "Take boar's grease, brains of a boar, powder of washed earth worms, red sanders, mummy and blood stone, of each one ounce; moss of a dead man's skull, not buried, one drachm; make an oyntment." All wounds are cured by this oyntment (provided the nerves and arteries be not hurt) thus: "Anoint the weapon that made the wound daily once, if there be need, and the wound be great; otherwise, it will be sufficient to anoint it every other day. Where note, 1, that the weapon be kept in clean linen, and in a temperate heat, lest the patient be hurt; for if the dust fall, or wind blow upon it, or it be cold, the sick will be much tormented; 2. That if it be a stab, the weapon to be anointed towards the point descending; 3. If you want the weapon, take blood from the wound upon a stick, and use as if t were the weapon. Thus the toothache is cured by pricking the gums and anointing the instrument. It is to be noted that the chrystals of vitriol converted into a white powder by a gentle heat is that which is called the sympathetical powder, which cureth wounds by washing a bloody cloth in the water in which it is dissolved."

These "remedies" seem very ridiculous to most of us now, yet are we constantly coming in contact with people, every day, who are doing equally stupid and silly things, for the cure of disease. When we learn better than to consult clairvoyants, tape-worm doctors, traveling doctors, and cease swallowing the vile and perfectly inert decoctions known as patent medicines, it will answer for us to laugh at the absurd practices of our ancient and less intelligent relatives. After ages of experience, we are yet forced to confess that the practice of medicine is entirely experimental, and can almost say, "I never took a remedy, but I've had lots of physic."

